# Cryo-EM reveals the conformation of a substrate analogue in the human 20S proteasome core

**DOI:** 10.1038/ncomms8573

**Published:** 2015-07-02

**Authors:** Paula C.A. da Fonseca, Edward P. Morris

**Affiliations:** 1 MRC Laboratory of Molecular Biology, Francis Crick Avenue, Cambridge CB2 0QH, UK; 2 Division of Structural Biology, The Institute of Cancer Research, London SW3 3RP, UK

## Abstract

The proteasome is a highly regulated protease complex fundamental for cell homeostasis and controlled cell cycle progression. It functions by removing a wide range of specifically tagged proteins, including key cellular regulators. Here we present the structure of the human 20S proteasome core bound to a substrate analogue inhibitor molecule, determined by electron cryo-microscopy (cryo-EM) and single-particle analysis at a resolution of around 3.5 Å. Our map allows the building of protein coordinates as well as defining the location and conformation of the inhibitor at the different active sites. These results open new prospects to tackle the proteasome functional mechanisms. Moreover, they also further demonstrate that cryo-EM is emerging as a realistic approach for general structural studies of protein–ligand interactions.

## Abstract (web summary)

The proteasome is a highly regulated complex fundamental for cell homeostasis and a target for cancer therapy. Here the authors use cryo-EM and single-particle analysis to obtain a detailed map of the interactions between each active sites of the core 20S proteasome and the irreversible inhibitor AdaAhx_3_L_3_VS.

## Graphical abstract

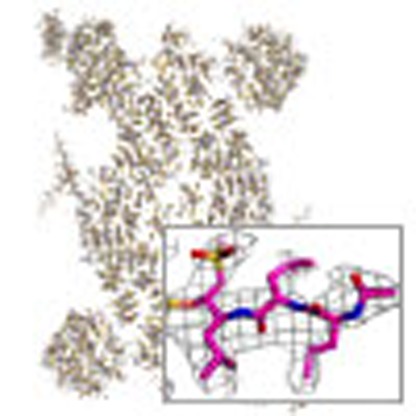

The proteasome comprises a 20S proteolytic core that in eukaryotes is formed from seven α and seven β subunits arranged in pseudo-seven-fold rings, two copies of which stack into a barrel shaped α_7_β_7_β_7_α_7_ assembly of about 750 kDa. The proteolytic active sites correspond to the amino-terminal (N-terminal) Thr residues of the mature β1, β2 and β5 subunits. Each of the active sites has distinct specificities, and variants of these are found in the interferon γ induced immunoproteasome, where the active subunits are replaced by their βi variants. Likewise, variants are also found in cortical thymic epithelial cells, where the βt5 thymoproteasome subunit is expressed. The crystal structure of a 20S proteasome core was first described for an archaeal counterpart^1^ and subsequently determined for yeast^2^ and constitutive bovine^3^ complexes. More recently, the crystal structures of both constitutive and immunoproteasome 20S cores from mouse have been reported^4^. These structures revealed the proteolytic sites enclosed within an inner chamber with very restricted access. Accordingly, the 20S core has limited proteolytic activity against smaller peptides and unfolded proteins^5^, and full activation requires the binding of regulatory complexes to the α rings at the ends of the barrel shaped core. Such regulatory complexes include the 19S regulatory particles, which bind to the 20S core to form the 26S proteasome and recruit fully folded protein substrates specifically tagged for degradation by ubiquitination^6^.

The proteasome is a well-established target for cancer therapy due to its critical role in the highly regulated removal of cell cycle regulators and to its inhibition leading to apoptosis, primarily of fast proliferating cells^7^. Bortezomid was the first inhibitor of the 20S core proteolytic activity to be approved for therapeutic usage against multiple myeloma, and a second-generation inhibitor, carfilzomib, is now used in the treatment of relapsed and refractory multiple myeloma^8^. In addition to the development of better anticancer drugs, proteasome inhibitors are being explored for other therapeutic usages including as anti-inflammatory and antiviral agents. However, due to the potential cytotoxic effects associated with 20S core inhibition, there is a great demand for the development of inhibitors with higher specificity. X-ray crystallography has been critical in determining the modes of interaction of inhibitors with the 20S core and to help the rational design of better therapeutic drugs^9^,^10^. However, these studies require co-crystallization of protein in the presence of inhibitor or soaking preformed protein crystals in an inhibitor containing solution. In either case, the experimental conditions must preserve the integrity of the protein crystals. These are not always ideal for the protein–ligand interactions and imply ligand binding under a non-physiological environment, where ligand accessibility may be modulated by steric restraints imposed by crystal contacts. With recent developments in the field of structural electron microscopy, including the availability of direct electron detectors, it is now possible to determine the structure of protein complexes by cryo-EM and single-particle analysis at resolutions that until recently have been attained only by crystallography or NMR^11^. Here we explore the use of cryo-EM as an emerging tool for the study of protein–ligand interactions, focusing on the structure of the human 20S proteasome core bound to an inhibitor.

## Results

### Cryo-EM structure of human 20S core with an inhibitor bound

**Figure 1:**
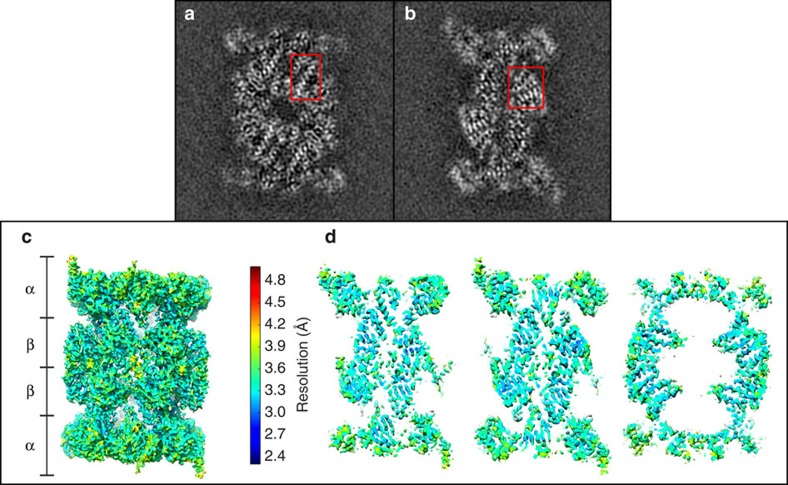
Cryo-EM map of the human 20S–AdaAhx_3_L_3_VS complex. (**a**,**b**) Individual sections 1 Å thick of the 3D map, as determined by the 3D reconstruction algorithm, are represented as grey-scale. Regions showing the pattern of α-helical secondary structure (**a**) and the separation of sheet forming β strands (**b**) are boxed. (**c**) Surface representation and (**d**) sections of the map surface rendered and colour coded according to local resolution as determined using ResMap^15^.

**Figure 2:**
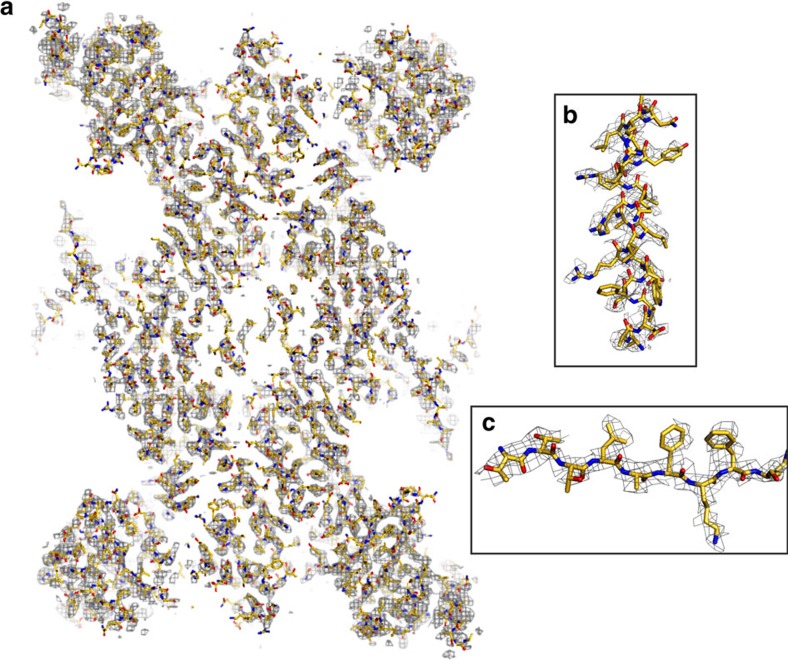
Mesh representation of the map of the human 20S–AdaAhx_3_L_3_VS complex, Fourier low-pass filtered to 3.4 Å, with built protein coordinates. (**a**) Section of the map showing the global agreement between the map densities and the coordinates of the 20S proteasome complex. Close-up representations of an α-helix (**b**) and a β strand (**c**) are shown, illustrating substantial recovery of side-chain information.

We determined the structure of the human 20S proteasome core bound to the inhibitor adamantaneacetyl-(6-aminohexanoyl)_3_-(leucyl)_3_-vinylmethyl-sulfone (AdaAhx_3_L_3_VS)^12^ by cryo-EM and single-particle analysis. The high quality of the final map can be effectively appreciated when viewed as individual sections 1 Å thick in grey-scale representation (Fig. 1a,b). Here protein density clearly stands out against a very smooth and clean background. Furthermore, close inspection allows immediate identification of the twist of α-helices (Fig. 1a) and reveals the clear separation of sheet forming β-strands (Fig. 1b). It should be noted that the map displayed in this way is obtained directly from the reconstitution algorithm, with no further filtering, sharpening or masking. In agreement with the clear recovery of structural detail in the grey-scale sections (Fig. 1a,b), the map allows unambiguous identification of the protein backbone. The model of the human 20S proteasome core (Fig. 2) was built based on the crystal structure of the mouse constitutive apo 20S core^4^. It reveals a conformational rearrangement from the starting model to the final human 20S–AdaAhx_3_L_3_VS complex resembling that previously described between the structure of apo and ligand bound 20S core complexes determined by X-ray crystallography^13^. The final human 20S–AdaAhx_3_L_3_VS model was checked for geometry, close contacts and bond parameters using MolProbity^14^ (Supplementary Fig. 1a). The quality of the model serves as clear indication that during its building there is no over fitting into noise in the EM map, as this would readily lead to poor MolProbity statistics. The resolution of the cryo-EM map can therefore be assessed by Fourier shell correlation against a density map generated from the coordinates of the molecular model yielding a value of about 3.5 Å (Supplementary Fig. 1b). Furthermore, an estimate of local resolution^15^ assigns the majority of the map voxels to a 3.3–3.8 Å resolution range (Fig. 1c,d and Supplementary Fig. 1c), which is consistent with the level of detail observed (Figs 1 and 2). In agreement with the resolution estimate, the map of the human 20S–AdaAhx_3_L_3_VS complex shows good resolution of most side chains (Fig. 2), with a better resolution observed in the protein interior where they are stabilized by intraprotein contacts and steric restrains. The reduced visibility of side chains at the protein surface appears to be related to solvent exposure rather than distortions due to contacts with the support carbon or with the air water interface, as similar effects are observed for exposed residues both at the outer surface and in the solvent filled interior cavities of the 20S core (Figs 1c,d and 2).

### Conformation of AdaAhx_3_L_3_VS at the different active sites

**Figure 3:**
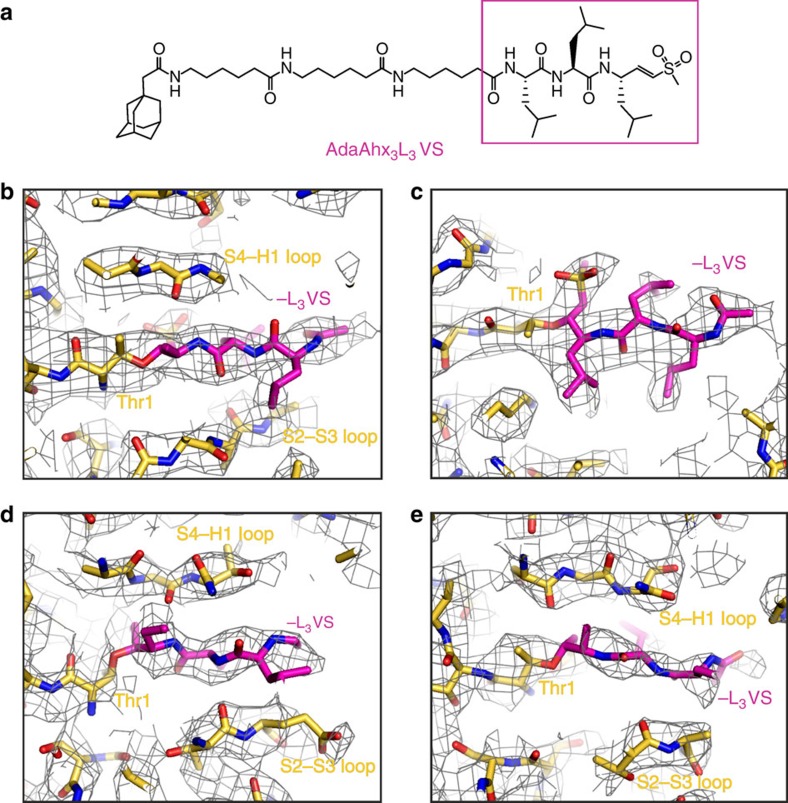
The ligand-binding pockets at the three 20S proteasome core active sites. (**a**) Structural formula of the inhibitor AdaAhx_3_L_3_VS. The map of the human 20S–AdaAhx_3_L_3_VS complex, Fourier low-pass filtered to 3.4 Å, is shown as a mesh (**b**–**e**). Clear densities are seen extending from the N-terminal Thr residues of the β5 (**b**,**c**), β2 (**d**) and β1 (**e**) subunits that are consistent with the -L_3_VS moiety of the AdaAhx_3_L_3_VS molecule, with the vinyl sulfone group and the side chains of the three leucine residues shown clearly resolved at the β5 site (**c**).

AdaAhx_3_L_3_VS (Fig. 3a) is a highly potent proteasome inhibitor that irreversibly binds all the 20S core proteolytic active sites and can be modified to serve as a proteasome label and as a reporter of proteasome inhibition both *in vitro* and *in vivo*^12^,^16^. The vinyl sulfone class of 20S core inhibitors act by covalently modifying the proteolytic active N-terminal Thr residues^17^. In the cryo-EM map of the 20S–AdaAhx_3_L_3_VS complex, densities are clearly recovered extending from the catalytically active residues of the β1, β2 and β5 subunits (Fig. 3b–e), with no analogous densities visible at the non-proteolytic subunits. In each case, this density is flanked by the loop between the β strands S2 and S3 and that linking the β strand S4 and the α helix H1 of the respective active subunit, in a similar manner to other 20S core inhibitors^4^,^18^,^19^. The density extending from the N terminus of the β5 subunit can be used to unambiguously build the L_3_VS moiety of the inhibitor (Fig. 3a). Here the vinyl sulfone group and the three leucine side chains are clearly resolved and arranged on alternate sides of the backbone in an extended near planar conformation (Fig. 3b,c). The remaining Ada and linker components of the inhibitor are only partially resolved, most likely due to conformational variability, and were therefore not modelled. The density extending from the N terminus of the β2 subunit is also consistent with an extended near planar conformation of the L_3_VS groups (Fig. 3d). Here, however, while the densities for the vinyl sulfone group and peptide backbone are well recovered, only partial density is found for the leucine side chains. A similar interpretation of the density extending from the N terminus of β1 can be made, although this is the least defined of the three inhibitor sites (Fig. 3e). Better visibility of the inhibitor densities at the β5 active site suggests higher occupancy compared with the β1 and β2 subunits. Accordingly, *in vitro* assays using purified mammalian 20S cores revealed that AdaAhx_3_L_3_VS inhibits the chymotryptic activity associated with the β5 subunit with higher potency than the activities associated with the β1 and β2 subunits^12^.

## Discussion

We present the structure determined by cryo-EM of the human 20S proteasome core bound to an inhibitor (AdaAhx_3_L_3_VS), at a resolution of about 3.5 ÅÅ, in which the protein backbone of each of its subunits is clearly identified as well as most of the side chains. Within the structure, AdaAhx_3_L_3_VS is clearly identified bound to each of the proteolytic sites of the 20S core. These results can be compared with recent structures of the archaeal apo 20S proteasome core, also determined by cryo-EM and single-particle analysis at resolutions of 3.3 Å (ref. 20) and 2.8 Å (ref. 21). Although related, the archaeal and the eukaryotic 20S proteasome cores differ substantially in their level of complexity. The archaeal 20S core is a D7 structure made up of seven-fold symmetric rings of homo–heptameric α and β subunits. The resulting 14-fold symmetry of this complex and its stability make it a very good model protein complex to test cryo-EM and image analysis procedures, due to the potential for symmetry-based averaging. In contrast, the eukaryotic 20S core has seven distinct α and β subunits. It conforms to lower C2 symmetry and, accordingly, the scope for internal averaging is reduced by a factor of 7. Furthermore, since in the archaeal 20S proteasome core all proteolytic active sites are identical, the analysis of this complex offers limited scope to explore and understand the selective targeting of the three individual proteolytic activities of the human 20S core complex. Consequently, the analysis of the archaeal complex in the presence of inhibitors would have substantially less relevance to drug development than the analysis of eukaryotic complexes. More broadly, the nature of the human 20S core is much more typical of a protein drug target, the majority of which conform to low symmetries, making it a good example to demonstrate the emerging potential of cryo-EM and single-particle analysis for the study of protein–ligand interactions and for structure-based drug design.

**Figure 4:**
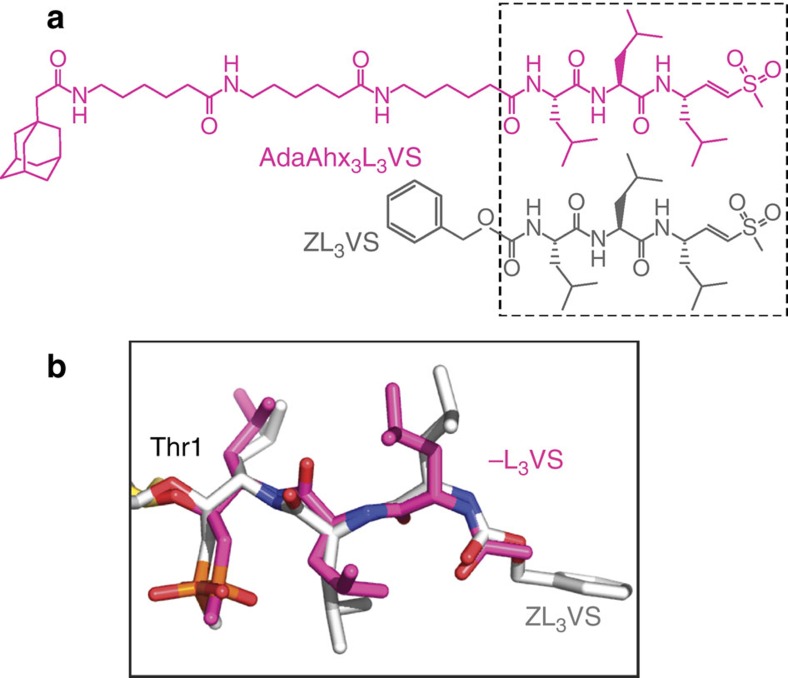
Conformations of the -L_3_VS moiety of AdaAhx_3_L_3_VS and ZL_3_VS bound to the 20S proteasome core. (**a**) Structural formulae of AdaAhx_3_L_3_VS and ZL_3_VS with their -L_3_VS moiety boxed. (**b**) The -L_3_VS moiety of the AdaAhx_3_L_3_VS molecule (in magenta) at the human 20S proteasome core β5 subunit active site is shown with superimposed coordinates for ZL_3_VS (grey), as found in a yeast 20S proteasome core crystallographic structure^19^.

Structure-based drug design has been extensively used for the development of improved 20S core inhibitors and until now these studies involved X-ray crystallography of 20S-inhibitor complexes. The closest of these inhibitors to the AdaAhx_3_L_3_VS used here is carboxybenzyl-(leucyl)_3_-vinyl-sulfone (ZL_3_VS), which was recently found binding only to the β5 subunit in a structure of the yeast 20S core^19^. Both compounds share the proteasome active site binding L_3_VS motif (Fig. 4a). By superimposing the structure of the ZL_3_VS bound to the yeast 20S core onto that of the AdaAhx_3_L_3_VS bound to the human complex, it is clear that both share a similar binding conformation (Fig. 4b). The densities extending from the N terminus of the β1 and β2 subunits of the human complex also indicate an extended conformation of the L_3_VS motif of the inhibitor, and their arrangement stacked within corresponding adjacent loops (Fig. 3d,e) seems to indicate a similar binding conformation of the L_3_VS motif at all three of the 20S core active sites. Furthermore, while in the yeast 20S–ZL_3_VS complex ZL_3_VS was found binding only to the β5 subunit, biochemical studies have shown that ZL_3_VS inhibits all three proteolytic activities of the 20S core. However, a somewhat stronger inhibition was observed for the chymotryptic activity of the β5 subunit^17^. The crystals of the yeast 20S–ZL_3_VS complex were obtained by soaking preformed protein crystals with an inhibitor solution^19^, under conditions that must preserve the crystals' integrity. However, for activity studies, proteasome samples are incubated in solution at 37 °C in the presence of inhibitor, under optimal conditions for protein–ligand interactions^17^. This seems to indicate that the absence of inhibitor at the β1 and β2 active sites in the crystal structure of the yeast 20S–ZL_3_VS complex may be a consequence of the experimental conditions used. On the other hand, in the closer to physiological conditions used for the cryo-EM sample preparation presented here, where the human 20S core was incubated in solution in the presence of AdaAhx_3_L_3_VS for 1 h at 37 °C, the inhibitor binds to all three of the active sites in a manner consistent with the results obtained from the *in vitro* activity studies using purified 20S core^12^.

Overall, our results illustrate the emerging potential of using cryo-EM and single-particle analysis to study the protein–ligand interactions and structure-based drug design, while opening new opportunities for the study of detailed protein functional mechanisms. Together with the recent report of the structure of the *Plasmodium falciparum* 80S ribosome bound to an anti-protozoan drug^22^, we show that cryo-EM can be used to visualize small ligands bound to protein complexes. Moreover, the results presented here also demonstrate the advantages of using cryo-EM for such studies, with results consistent with activity assays, as it allows the analysis of complexes rapidly frozen from solution and thus under conditions closer to physiological compared with other methods of structural determination.

## Methods

### Sample preparation

The human 20S proteasome core sample and AdaAhx_3_L_3_VS were purchased from Enzo Life Sciences. The 20S proteasome core was diluted in 50 mM Tris-HCl, pH 7.5, 5 mM MgCl_2_ and 1 mM dithiotreitol to a concentration of 0.14 μM (0.1 mg ml^−1^) and incubated in the presence of 10 μM AdaAhx_3_L_3_VS for 1 h at 37 °C.

### Cryo-EM

Samples were applied onto thin layers of carbon freshly floated from mica and supported by 1.2/1.3 Quantifoil electron microscope grids. They were then flash frozen by plunging into liquid ethane using a Vitrobot (FEI), operated at 22 °C and 95% humidity, with a 2.5 s blot time. During their preparation, the grids were rendered hydrophilic by glow discharging. It was observed that the glow discharging of the grids in air causes the 20S proteasome core to orient predominantly with its long axis perpendicular to the support carbon. To get the random side views required for data completeness in the three-dimensional (3D) reconstructions, it was necessary to glow discharge the grids in the presence of pentylamine (Supplementary Fig. 2). The grids were transferred into a FEI Titan Krios microscope operated at an acceleration voltage of 300 keV, a nominal magnification of × 75,000 (yielding a calibrated sampling of 1.04 Å per pixel at the image level) and a range of 1.7–3.0 μm underfocus. Images were recorded with EPU software using a Falcon II direct electron detector, with 1 s exposure time, giving 17 individual frames captured at a rate of 0.056 s per frame and each corresponding to an electron dose of 2.8 e^−^ Å^−2^ (Supplementary Fig. 3).

### Image processing

The strategy for image processing was based on that previously described for the structure of the human 26S proteasome^23^, but taking advantage of the new instrumentation and image recording protocol. For each exposure, the sum of all frames captured was used to carefully screen for optimal ice thickness and image stability by assessing the isotropic recovery of Thon rings in their power spectra. The power spectra of the images selected for analysis had isotropic Thon rings directly observed to 4 Å or better. Using these criteria, 720 images were selected for further processing from a total of 960 exposures recorded, all of these acquired from a single cryo-EM grid and during a single cryo-EM session. The sum of all frames recorded for each of the exposures selected for further processing was also used to determine the image defocus and astigmatism parameters, using the Tigris programme findctf, and for particle selection, which was performed automatically in Relion^24^. This was followed by careful inspection of all particles selected and manual removal of false positives and addition of false negatives, resulting in a data set of 76,500 particles. Further image processing was done using the sum of frames 3–10 of each exposure, corresponding to a total accumulated electron dose of 28 e^−^ Å^−2^. This selection aimed at maximum specimen stability and optimal signal-to-noise in the resulting images for accurate determination of the particle orientations, while avoiding excessive radiation damage. In this selection, the first two frames captured during exposure, corresponding to the initial accumulated dose of 5.6 e^−^ Å^−2^, were excluded as it is at the start of the exposure that the most prominent beam induced particle movements are observed^25^. As a consequence of our data collection protocol, for each exposure the selection of recorded frames used for high-resolution analysis was effectively acquired within 0.45 s. Within this short time, the individual particle images showed negligible translational displacement and hence it was not judged necessary to further align these frames. The phases of the resulting images were corrected for the contrast transfer function by phase flipping with the Tigris programme flipctf, using the image defocus and astigmatism parameters determined using the full exposures as described above. Selected particle images were then extracted into 256 × 256 pixel boxes. A model derived from the coordinates of the bovine constitutive apo 20S proteasome core, as fitted into the 3D map of the human 26S proteasome^23^ and Fourier low-pass filtered to 20 Å, was used as an initial reference for single-particle analysis of the human 20S proteasome core in the apo state (data not shown). The resulting map, again low-pass filtered to 20 Å, was used as a starting reference for the analysis of the human 20S–AdaAhx_3_L_3_VS complex. The single-particle analysis refinement routine, performed using C2 symmetry, consisted of rounds of alignment and Euler angle assignment using the programme AP SH of the Spider software package^26^, 3D reconstruction and forward projection. 3D reconstructions and forward projections were performed using the locally developed icr3d and icr3dpro programs implemented in Tigris. The 3D reconstruction algorithm included correction for the amplitude component of the contrast transfer function. The Tigris software package is publicly available at sourceforge.net. The surface representations of the final map, as determined by the 3D reconstruction algorithm without further sharpening, Fourier filtering or masking, shown in Fig. 1c,d were created with UCSF Chimera^27^. They were surface rendered and colour coded according to local resolution as determined by ResMap^15^.

### Molecular modelling

The model of the human 20S proteasome core was built based on the X-ray crystal structure of the mouse constitutive apo 20S proteasome core^4^ using real-space refinement in Coot^28^ and Phenix^29^. The model generated in this way was checked for geometry, close contacts and bond parameters using MolProbity^14^ (Supplementary Fig. 1a), with the statistics obtained consistent with those for models determined by X-ray crystallography at similar resolutions. At the stage of model building, the scaling of the 3D map of the human 20S–AdaAhx_3_L_3_VS complex was tested, and model building using a sampling of 1.04 Å per pixel resulted in the most favourable protein coordinate MolProbity scores, indicating accuracy in the sampling used. Models for the individual copies of the -L_3_VS moiety of the AdaAhx_3_L_3_VS molecule, at each of the 20S proteasome core active sites, were built in using Coot. The mesh representations of the final map showing the model of the human 20S proteasome core (Figs 2 and 3) were created using the PyMOL Molecular Graphics System. For these representations, the map was sharpened with a B-factor of −50 and Fourier low-pass filtered to 3.4 Å.

## Additional information

**Accession codes**: The cryo-EM map (as used to prepare Figure 1) and the atomic coordinates have been deposited in the Electron Microscopy Data Bank and Protein Data Bank with the accession codes EMD-2981 and 5a0q, respectively.

**How to cite this article:** da Fonseca, P. C. A & Morris, E. P. Cryo-EM reveals the conformation of a substrate analogue in the human 20S proteasome core. *Nat. Commun.* 6:7573 doi: 10.1038/ncomms8573 (2015).

## Supplementary Material

Supplementary Information Supplementary Figures 1-3 and Supplementary References
