# Supplementary material for: Cryo-EM reveals the conformation of a substrate analogue in the human 20S proteasome core
Source: Nat Commun. 2015 Jul 2;6:7573. doi: 10.1038/ncomms8573 (PMC4506541; doi:10.1038/ncomms8573)
Supplement: Supplementary Information — Supplementary Figures 1-3 and Supplementary References [file ncomms8573-s1.pdf]

a

MolProbity results		
**All Atom Contacts**		
MolProbity clashscore	30.65	(81th percentile, >3.0 Å)
**Protein Geometry**		
Poor rotamers	0.69 %	
Ramachandran plot		
favored	95.23 %	
allowed	4.40 %	
outliers	0.37 %	
MolProbity score	2.30	(99th percentile, 3.5±0.25 Å)
Cβ deviations >0.25Å	0.23 %	
Bad backbone bonds	0.28 %	
Bad backbone angles	0.76 %	

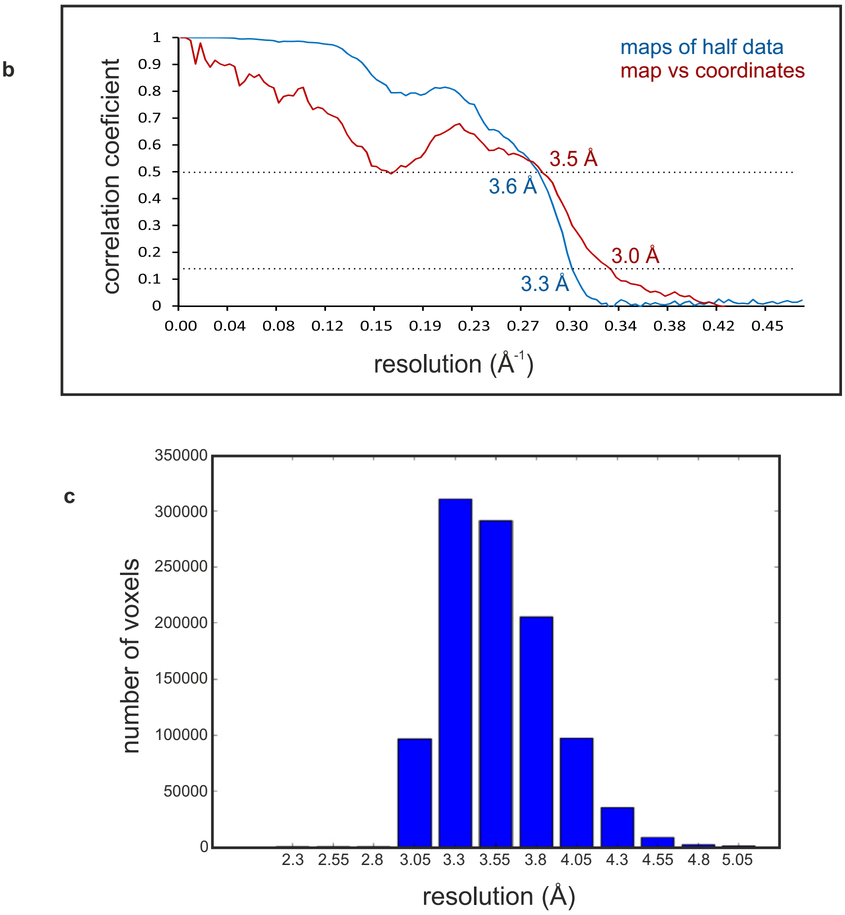

Supplementary Figure 1. Evaluation of the molecular model of the human 20S proteasome core and resolution estimate for the cryo-EM map of the 20S proteasome core. **(a)** Evaluation of the model of the human 20S proteasome core using MolProbity [1]. (**b**) Resolution estimate of the cryo-EM map by Fourier shell correlation. The curves correspond to the correlation obtained against the model of the human 20S proteasome core (red) and the correlation between two halves of the data (blue). The resolution was estimated from the curve against the model where the 0.5 correlation coefficient criterion[2] yields an estimate of 3.5 Å. **(c)** Distribution of estimated local resolution within the cryo-EM map calculated with ResMap [3], with the majority of voxels assigned within the resolution range 3.3-3.8Å.

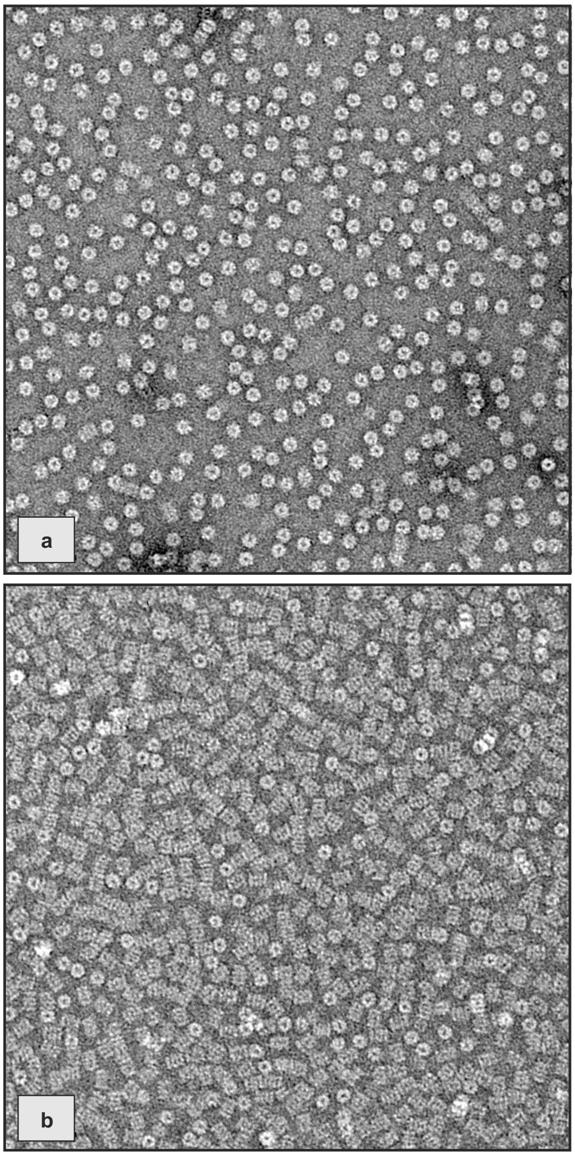

Supplementary Figure 2. The effect of glow discharge in the presence of pentylamine on the orientation of the human 20S proteasome core complexes on carbon coated electron microscope grids. Fields of negatively stained 20S proteasome molecular images applied to carbon coated grids prepared with (**a**) standard (air) glow discharge and (**b**) glow discharge in the presence of pentylamine. Top views predominate in (**a**) while randomly oriented side views predominate in (**b**).

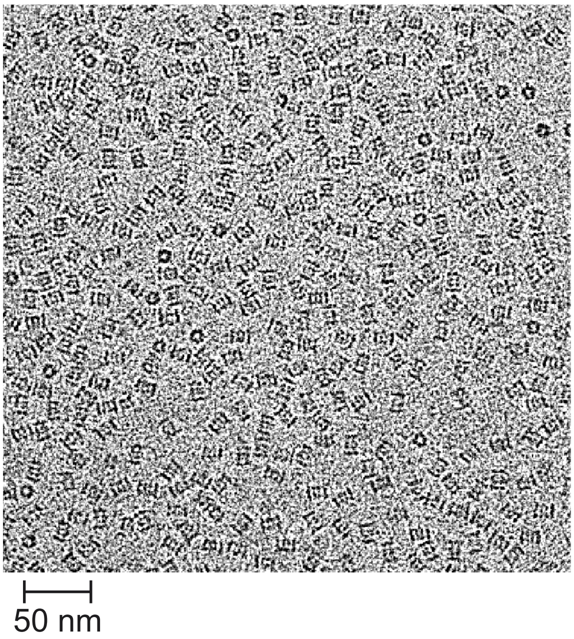

Supplementary Figure 3. Cryo-EM image of human 20S proteasome on pentylamine glow discharged carbon coated grid. Image recorded at 3μm underfocus with an electron dose of 50 e^{-} $Å^{-2}$. The high signal:noise ratio and the high level of detail in the molecular images of the 750 kDa complex can be ascribed to optimal ice thickness and the favourable characteristics of the imaging conditions.

Supplementary References:

1 Chen, V. B. *et al.* MolProbity: all-atom structure validation for macromolecular crystallography. *Acta Crystallographica Section D* **66**, 12-21 (2010).

2 Rosenthal, P. B. & Henderson, R. Optimal determination of particle orientation, absolute hand, and contrast loss in single-particle electron cryomicroscopy. *J Mol Biol* **333**, 721-745 (2003).

3 Kucukelbir, A., Sigworth, F. J. & Tagare, H. D. Quantifying the local resolution of cryo-EM density maps. *Nat Methods* **11**, 63-65 (2014).
